# A Novel Approach for Acoustic Signal Processing of a Drum Shearer Based on Improved Variational Mode Decomposition and Cluster Analysis

**DOI:** 10.3390/s20102949

**Published:** 2020-05-22

**Authors:** Changpeng Li, Tianhao Peng, Yanmin Zhu

**Affiliations:** School of Mechanical Engineering, Anhui University of Science & Technology, No168 Taifeng Road, Huainan 232001, China; 2017200317@aust.edu.cn (C.L.); ymzhu@aust.edu.cn (Y.Z.)

**Keywords:** drum shearer, acoustic signal, variational mode decomposition, parameter optimization, particle swarm optimization, cluster analysis

## Abstract

During operation, the acoustic signal of the drum shearer contains a wealth of information. The monitoring or diagnosis system based on acoustic signal has obvious advantages. However, the signal is challenging to extract and recognize. Therefore, this paper proposes an approach for acoustic signal processing of a shearer based on the parameter optimized variational mode decomposition (VMD) method and a clustering algorithm. First, the particle swarm optimization (PSO) algorithm searched for the best parameter combination of the VMD. According to the results, the approach determined the number of modes and penalty parameters for VMD. Then the improved VMD algorithm decomposed the acoustic signal. It selected the ideal component through the minimum envelope entropy. The PSO was designed to optimize the clustering analysis, and the minimum envelope entropy of the acoustic signal was regarded as the feature for classification. We then use a shearer simulation platform to collect the acoustic signal and use the approach proposed in this paper to process and classify the signal. The experimental results show that the approach proposed can effectively extract the features of the acoustic signal of the shearer. The recognition accuracy of the acoustic signal was high, which has practical application value.

## 1. Introduction

A drum shearer is a key piece of equipment in a fully mechanized coal face. Improvement to its automation level is critical to the efficient mining and safe production of the entire coal mining face [1]. Research on coal and rock identification technology has promoted drum shearer automation development. For instance, Bessinger used a γ-ray device to measure the thickness of the residual coal seam in the roof of a coal mine [2]. This method is restricted by environmental factors. Fan utilized the pressure change of the cylinder in the working process of the drum shearer as the characteristic signal and extracted the mean square error of the signal as the characteristic vector for the identification of coal and rock [3]. However, there are too many factors affecting the pressure change, and the recognition results are more volatile. Moreover, Sun took advantage of image analysis technology to extract texture features from coal and rock for classification and recognition using a gray level co-occurrence matrix [4]. Zhang analyzed the differences between the thermal infrared image characteristics and the transient flash temperature value during the cutting process [5]. Nevertheless, the dust in the coal mine affects the image collection. By installing an acoustic sensor on the rocker arm of the shearer, Xu used ensemble empirical mode decomposition (EEMD) and a neural network to identify coal and rock, which is hard to avoid using numerous computer simulations to determine method parameters [6,7]. In addition, Si proposed an improved method of coal rock recognition based on the multi-scale fuzzy entropy and support vector machine (SVM) by extracting the vibration signal of the shearer’s rocker arm and combining the concepts of multi-scale entropy and fuzzy entropy [8]. Song established the feature data set of coal rock recognition by collecting the vibration and acoustic signals. He then proposed a classification method for combining the minimum enclosing balls (MEB) and SVM [9,10]. However, they ignore the effects of noise. Li decomposed the acoustic emission signal by using the Coiflet wavelet transform (WT) to gain multiple eigenvector groups. Afterward, he combined a multi-resolution fusion method and SVM to recognize coal and rock [11]. Wang proposed a method of multi-sensor information fusion combining vibrations, acoustic emission, a cutting current, and infrared thermal imaging to identify coal and rock [12]. The recognition method of multi information fusion increases the computation. When shearers are functional, the experienced operator will adjust the height of the drum according to the cutting sound to carry out the coal cutting. Physiological research has shown that to some extent, the human ear has the characteristics of localization and time-frequency decomposition [13,14]. Therefore, in various complicated environments, the human ear can accurately capture necessary acoustic signals. The acoustic signal can recognize coal rock due to its characteristics, such as easy gaining and non-contact measurement. The research on acoustic signal processing was also constantly developing. In the last century, many have used a fast Fourier transform to interpret the acoustic signal. Nevertheless, it is considered a non-stationary signal. As a result, the traditional Fourier transform was not applicable.

The non-stationary signal has poor stability and strong randomness, which can cause physical information confusion [15]. Hence, signal processing methods, such as wavelet transform (WT) [16], empirical mode decomposition (EMD) [17], EEMD [18], and the local mean decomposition (LMD) [19] have developed rapidly. The WT resolves the original signal into the accumulation of multiple wavelets in the form of a window function. Thus, WT has the advantage of having multi-resolution. Yan introduced the application of the classical WT, continuous WT, discrete WT, and the wavelet packet transform (WPT) in the fault diagnosis of rotating machinery [20]. However, selecting wavelet basis functions and decomposition levels restricts the application of WT. There will be some differences in the analysis results obtained by selecting different basis functions and decomposition levels. This signal decomposition method is easy to cause information leakage. To some extent, WT is not a real adaptive signal processing method. Huang adaptively decomposed the signal into several intrinsic mode functions (IMF) by EMD and realized the time-frequency analysis of the signal with Hilbert transform [21]. Moreover, Klionskiy explored the use of EMD for signal denoising [22]. Different from EMD, in order to suppress the mode confusion in the decomposition, EEMD adds Gaussian noise signals to the processing [23]. Zhang utilized EEMD to handle the vibration signals of motor bearings, took the replacement entropy of the IMF as the eigenvector, and combined the eigenvector with a SVM to classify the fault types [24]. Whether it is EMD or EEMD, issues remain, such as the end effect or mode confusion in the application. The LMD can solve irregular signals and even multiple product function components with a physical meaning. Nevertheless, for the signal with a similar frequency, it cannot separate effectively [25].

Thus, Dragomiretskiy and Zosso first proposed a new mode decomposition method: variational mode decomposition (VMD), which can adapt to signal processing [26]. The VMD method is primarily based on the Wiener filter, Hilbert transform, and frequency mixing. In the variational framework, this method iteratively searches for the optimal solution of the mode and continuously updates each mode function and the center frequency to obtain several mode functions with a particular bandwidth. It is a completely non-recursive signal decomposition method. Compared with other mode decomposition, VMD has an entirely theoretical basis. It has significant advantages in separating similar frequencies, suppressing mode confusion, and avoiding the end effect [27]. However, the accuracy of the signal processed by the VMD often depends on the preset parameters, which lacks adaptability. In order to conquer this drawback, numerous researches have been conducted. Li proposed an independence-oriented VMD method based on peak searching and similarity principle [28]. With this method, the appropriate number of components can be determined, but it ignores the influence of other parameters. Based on a genetic algorithm (GA), Yan introduced an improved VMD method to obtain optimal parameters [29]. Nevertheless, the objective function of this method is partial, and it may cause the loss of information. Yi calculated the correlation between the mode components and the original signal after signal decomposition and used the ratio between the mean value and variance of the correlation as the fitness function of particle swarm optimization (PSO) to improve the VMD method [30]. Although this method can obtain the appropriate parameter value, it requires too much calculation. In addition, Zhang used the grasshopper optimization algorithm to propose a parameter-adaptive VMD method [31]. Yan designed an improved VMD method which uses a cuckoo search algorithm to obtain ideal parameters [32]. Zheng used adaptive differential evolution to determine the parameters of the VMD method [33]. In fact, the parameters of VMD can be adaptively determined by using meta-heuristic algorithms, but the appropriate fitness function needs to be selected first.

Cluster analysis refers to the classification of any sample category or other prior knowledge in a batch of samples, which are based on the characteristics of the samples. It classifies the same or similar features into one class by some similarity measurement method [34]. Clustering analysis is often used widely in pattern recognition, machine learning, and other fields. For the sensitivity of the method, Ahmad extended the k-harmonic mean clustering algorithm to mix data sets and compared the clustering effect of the algorithm in pure classification data and mixed data through various experiments [35]. In addition, Baraldi combined the unsupervised fuzzy C-means (FCM) algorithm with clustering analysis technology for fault diagnosis of nuclear turbines [36]. However, the algorithm relies heavily on the selection of the membership matrix and the initial center point. By continuously iterating to obtain the optimal objective function, the algorithm has a high time complexity. For better clustering effects and to solve issues that the algorithm is easy to fall into local optimization, intelligent optimization algorithms have been useful. Combined with the GA [37], ant colony optimization (ACO) [38], and PSO [39], cluster analysis has improved its global search ability significantly.

As a type of meta-heuristic algorithm, swarm intelligence optimization algorithm is a bionic algorithm based on population strategy. Inspired by the life processes of natural organisms, researchers have put forward a variety of swarm intelligence optimization algorithms and continue to supplement and improve upon them [40]. The main methods primarily include GA, ACO, and PSO. GA is a kind of evolutionary calculation that simulates the inheritance of biological genes. This method has strong convergence, good expansibility, and high universality. However, the algorithm relies on the setting of initial population parameters. ACO adopts a parallel strategy, which improves the reliability of the algorithm and also makes the algorithm have a better global optimization ability. Nevertheless, this algorithm is computationally intensive and requires a long solution time. PSO originates in the behavior of primitive biological groups, such as birds. Beginning with a random solution, an iterative approach can be used to find the ideal solution. This algorithm has the characteristics of a simple algorithm, high precision, fast convergence, and so on. For instance, Yahya took advantage of PSO to classify high-dimensional data [41]. Moreover, Malik used PSO to optimize the neural network for the prediction of building energy consumption [42]. Maleki determined the best parameters of a PV/wind/battery hybrid system by studying different PSO variant algorithms [43]. The research of PSO has been divided into theoretical research and engineering applications. Some scholars have studied the algorithm’s structure and performance improvement. More researchers have applied this algorithm to data classification, multi-objective optimization, fault diagnosis, and path planning.

On the basis of the aforementioned research, this paper proposes an approach for analyzing and processing the acoustic signal of the drum shearer. Two typical acoustic signals produced by drum shearer are selected for analysis. One is the acoustic signal of the transmission system driven by the motor in the rocker arm. Another is the acoustic signal of the hydraulic system powered by the electric machinery in the pump station. When the VMD algorithm interprets the collected acoustic signal, the parameters, such as model decomposition number *K*, penalty factor α, and the convergence condition must be preset. In this paper, the PSO algorithm searches and decides the best parameter combination of VMD. Considering the acoustic signal of shearer has periodic impact component, the peak factor of the envelope spectrum can represent the characteristics of the impact component. Set the local maximum envelope spectral peak factor as the fitness function of PSO. In the optimization process, the final result is determined by searching for the maximum fitness function value. Then, VMD, with optimized parameters, handles the acoustic signal. Calculate the envelope entropy of the component signal and select the minimum envelope entropy as the eigenvalue. Moreover, cluster analysis is used to classify the acoustic signal. Meanwhile, considering the clustering algorithm’s shortcoming of easily falling into the local limit value, the PSO iterative search for the ideal value is utilized to optimize the clustering algorithm.

The reminder of this paper is arranged as follows. In Section 2, the basic theory of VMD, PSO, and the clustering analysis algorithm are introduced. In Section 3, the parameter optimization process of VMD is described. In Section 4, a simulation signal is established, and the signal analysis was carried out using this approach. In Section 5, the building of the simulation experiment platform of the shearer and obtaining the acoustic signal of the drum shearer are detailed. Then, the acoustic signal is processed by the approach proposed in this paper, and the clustering analysis realizes the classification. Finally, in Section 6, some conclusions and prospects are summarized.

## 2. Basic Theory

### 2.1. Variational Mode Decomposition

#### 2.1.1. Basic Principles of VMD

In the VMD algorithm, the original signal *f(t)* was decomposed into *K* IMFs $u_{k}$ with a specific bandwidth. The center frequency of each IMF is reflected by $\omega_{k}$. The following defines the IMF as an amplitude modulation-frequency modulation signal:

(1) $$f(t)=\sum_{i=1}^{K}u_{k}(t)$$

(2) $$u_{k}(t)=A_{k}(t)\cos(\varphi_{k}(t))$$

where $A_{k}\;(t)$ is the instantaneous amplitude of $u_{k}\left(t\right)$, and $A_{k}\left(t\right)\;\geq \;0$. $\varphi_{k}\left(t\right)$ indicates the phase. $\varphi_{k}{(t)=\varphi }_{k}^{\prime }\left(t\right)$ reflects the instantaneous frequency, and $\varphi_{k}^{\prime }\left(t\right)\;>\;0$.

The Hilbert transform is applied to $u_{k\;}\left(t\right)$ to construct the analytic signal and obtain its one-sided spectrum:

(3) $$\left[\delta (t)+\frac{j}{\pi t}\right]\times u_{k}(t)$$

where δ (t) indicates a pulse function.

The exponential function $e^{{-j\omega }_{k}t}$ mixes the analytic signals of $u_{k\;}\left(t\right)$ and modulates the frequency spectrum of the IMF component to the corresponding fundamental frequency spectrum.

(4) $$\left[\left(\delta (t)+\frac{j}{\pi t}\right)\times u_{k}(t)\right]e^{-j\omega_{k}t}$$

To determine the norm of the square $L^{2}$ of the aforementioned demodulation signal gradient, estimate the signal width of each IMF component, and establish a constrained variational model, the following formula is used:

(5) $$\underset{\left\{u_{k}\right\},\left\{\omega_{k}\right\}}{\min}\left\{{\sum_{k}\left\|\partial_{t}\left[\left(\delta (t)+\frac{j}{\pi t}\right)\times u_{k}(t)\right]e^{-j\omega_{k}t}\right\|}_{2}^{2}\right\}$$

(6) $$s.t.\sum_{k}u_{k}=f(t)$$

where $\left\{u_{k}\right\}{=\{u}_{1},{\;u}_{2},\;\cdots {,\;u}_{K}\}$ indicates the *K* model components gained through decomposition. $\left\{\omega_{k}\right\}{=\{\omega }_{1}{,\;\omega }_{2},\;\cdots {\;\omega }_{K}\}$ reflects the frequency center of each component.

For the best solution of the aforementioned constrained variational model, it must become an unconstrained variational problem thereby introducing the Lagrange multiplier λ and quadratic penalty factor α. The augmented Lagrange function is:

(7) $$L\left(\left\{u_{k}\right\},\left\{\omega_{k}\right\},\lambda \right)=\alpha {\sum_{k}\left\|\partial_{t}\left[\left(\delta (t)+\frac{j}{\pi t}\right)\times u_{k}(t)\right]e^{-j\omega_{k}t}\right\|}_{2}^{2}+{\left\|f(t)-\sum_{k}u_{k}(t)\right\|}_{2}^{2}+\langle \lambda (t),f(t)-\sum_{k}u_{k}(t)\rangle$$

The alternating direction multiplier method solves the aforementioned variational problem. The center frequency and bandwidth of each IMF component alternately update to find the saddle point of the augmented Lagrange function. Using the Parseval/Plancherel Fourier isometric transform, the model component $u_{k}$ and the center frequency $\omega_{k}$ are obtained as follows:

(8) $${\overset{\wedge }{u}}_{k}^{n+1}(\omega )=\frac{\overset{\wedge }{f}(\omega )-\sum_{i\neq k}{\overset{\wedge }{u}}_{i}(\omega )+\overset{\wedge }{\lambda }(\omega )/2}{1+2\alpha {\left(\omega -\omega_{k}\right)}^{2}}$$

(9) $$\omega_{k}^{n+1}=\frac{\int_{0}^{\infty }\omega {\left|{\overset{\wedge }{u}}_{k}(\omega )\right|}^{2}d\omega }{\int_{0}^{\infty }{\left|{\overset{\wedge }{u}}_{k}(\omega )\right|}^{2}d\omega }$$

where ${\overset{\wedge }{u}}_{k}^{n+1}$ is the Wiener filter of the current residual $\overset{\wedge }{f}(\omega )\;-\sum_{i\neq k}{\overset{\wedge }{u}}_{i}\left(\omega \right)$. $\omega_{k}^{n+1}$ is the center frequency of the power spectrum of the current model function, and $\left|{\overset{\wedge }{u}}_{k}(\omega )\right|$ is the inverse Fourier transform.

#### 2.1.2. VMD Iterative Operation

1 Initialize $\left\{\overset{\wedge \;1}{u_{k}}\right\}$, $\left\{\overset{\wedge \;1}{\omega_{k}}\right\}$,$\overset{\wedge \;1}{\lambda }$, *n.*
2 Update $u_{k}$ and $\omega_{k}$ according to Formulas (8) and (9).
3 Preset the fidelity coefficient τ. Update λ according to Formula (10):
  (10) $${\overset{\wedge }{\lambda }}^{n+1}(\omega )\leftarrow \overset{\wedge \;n}{\lambda }(\omega )+\tau (\overset{\wedge }{f(}\omega )-\sum_{k}{\overset{\wedge }{u}}_{k}^{n+1}(\omega ))$$
4 Set the precision e > 0. Settle the criteria as Formula (11). If the condition is met, the iteration is terminated. Otherwise, return to step (2) and continue the loop.
  (11) $${{\sum_{k}\left\|{\overset{\wedge }{u}}_{k}^{n+1}(\omega )-{\overset{\wedge }{u}}_{k}^{n}(\omega )\right\|}_{2}^{2}/\left\|{\overset{\wedge }{u}}_{k}^{n}(\omega )\right\|}_{2}^{2}<e$$

### 2.2. Particle Swarm Optimization

The PSO algorithm is derived from the complex adaptive system. It is an optimization algorithm based on an iterative mode. Through cooperation and competition among individuals, PSO realizes the search for the ideal solution in a complex space. Every possible solution is expressed as a particle in the group. This particle has its own velocity vector and position vector, as well as a fitness determined by the objective function. All particles are flying in a complex space at a specific speed and are searching the global optimal value by seeking the current optimal value.

The algorithm assumes there is an *n*-dimensional target search space. *m* particles constitute the population ${S=\{\;S}_{1}{,\;S}_{2},\;\cdots {,\;S}_{m}\;\}$. Any particle *i* is located at $S_{i}{=\{S}_{i1}{,\;S}_{i2},\;\cdots {,\;S}_{im}\}$. The position of each particle *i* is a potential solution and particle *i* searches for new solutions by constantly adjusting its position $S_{i}$. $P_{id}$ describes the best solution found by particle *i*. $P_{gd}$ is the optimal solution currently searched for the entire particle population. The velocity of the *i*-th particle represented as $V_{i}=\left(\;v_{i1}{,\;v}_{i2},\;\cdots {,\;v}_{in}\;\right)$. When the population finds $P_{id}$ and $P_{gd}$, particle *i* updates its speed according to Formulas (12) and (13).

(12) $$v_{id}(t+1)=\omega v_{id}(t)+c_{1}r_{1}(t)(p_{id}-s_{id}(t))+c_{2}r_{2}(p_{gd}-s_{id}(t))$$

(13) $$s_{id}(t+1)=s_{id}(t)+v_{id}(t+1)$$

where $v_{id}\left(t+1\right)$ is the velocity of particle *i* in the *d*-th dimension of *t +* 1 iteration. To reduce the possibility of particle i flying out of the search space, the limited speed range was between $\left[{-v}_{max}{,\;v}_{max\;}\right]$. $v_{max}$ is the maximum velocity of particle *i*. *ω* is the inertia weight coefficient, which controls the influence of the front speed on the current velocity. A larger *ω* is helpful to the global search ability of PSO. In contrast, a smaller *ω* enhances the local search ability. $c_{1}$ and $c_{2}$ are learning factors and non-negative constants. $c_{1}$ adjusts the step length of particle *i* to an optimal position. The step length of the global optimal position direction of particle *i* is regulated by $c_{2}$. Appropriate $c_{1}$ and $c_{2}$ can accelerate the algorithm convergence and reduce local optimization. $r_{1}$ and $r_{2}$ are independent pseudo-random numbers, which distribute on [0,1], uniformly.

The basic steps of PSO are as follows:

1 Particle swarm initialization: set the initial velocity and initial position of each particle randomly.
2 The new position of each particle will be generated by the initial velocity and position.
3 Calculate the fitness of particles.
4 Compare the fitness value of arbitrary particles with the fitness value of the optimal position $P_{id}$ it experienced. If there exists a better fitness value, it will update.
5 Compare the fitness value of any particle with the fitness value of the optimal position $P_{gd}$ passed by the population. If there is a better fitness value, then $P_{gd}$ will be replaced.
6 Adjust the position and speed of particles according to Formulas (12) and (13).
7 If the ideal position is searched or the maximum number of iterations is reached, the optimization ends. Otherwise, repeat steps 3 to 6.

### 2.3. Clustering Analysis of PSO

Let the sample pattern set be ${R=\{R}_{i},\;i=1,\;2,\;\cdots ,\;N\}$. $R_{i}$ is the *n*-dimensional pattern vector, and cluster analysis is done to find a partition ${\varphi =\{\varphi }_{1}{,\;\varphi }_{2},\;\cdots {,\;\varphi }_{M}\}$, so that the sum of the total within-class scatter reaches the minimum value. The sum of the distances of various types of samples to the corresponding cluster centers can be acquired by the clustering criterion formula *J*:

(14) $$J=\sum_{j=1}^{M}\sum_{R_{i}\in \varphi_{j}}d\left(R_{i},\bar{R^{\left(\varphi_{j}\right)}}\right)$$

where $\bar{R^{{(\varphi }_{j})}}$ represents the center of the *j*-th cluster, and ${d(R}_{i},\;\bar{R^{{(\varphi }_{j})}}$) is the distance from the sample to the corresponding center. After deciding the clustering center, the clustering division can be determined by the nearest neighbor method. For a sample $R_{i}$, if the center $\;\bar{R^{{(\varphi }_{j})}}$ of the *j*-th cluster satisfies Formula (15), then $R_{i}$ belongs to the *j*-classification.

(15) $$d\left(R_{i},\bar{R^{\left(\varphi_{j}\right)}}\right)=\underset{l=1,2,\cdots M}{\min}d\left(R_{i},\bar{R^{\left(\varphi_{l}\right)}}\right)$$

When using PSO to solve clustering issues, each particle *i* is regarded as a feasible solution to form a particle swarm solution set. It assumes that the clustering issue with *M* clustering centers and the *n*-dimensional sample vector dimension is known. Any particle *i* can be determined by particle position, velocity, and fitness value. The fitness of particle *i* can be calculated per the following method.

1 The clustering division of particle *i* can be determined by the nearest neighbor method of Formula (15).
2 On the basis of the partitioned clusters, the cluster center recalculates. Then, count the total within-class scatter *J* according to Formula (14).
3 The fitness computing Formula (16) of particle *i* is as follows:
  (16) $$P_{fitness}=k/J$$
  where *k* is a constant that depends on the specific situation. The fitness value of particle *i* negatively correlates with the *J* value of the clustering partition.

## 3. Parameter Optimization of VMD

The influencing parameters of the VMD algorithm primarily include the number of components, *K*; penalty factor, α; discrimination accuracy, ε; and fidelity coefficient, τ. Constructing the multi-harmonic signal is as shown in Formula (17).

(17) $$x(t)=\sin(8\pi t)+\frac{1}{6}\cos(128\pi t)+\frac{1}{32}\sin(512\pi t)+\eta$$

**Figure 1:**
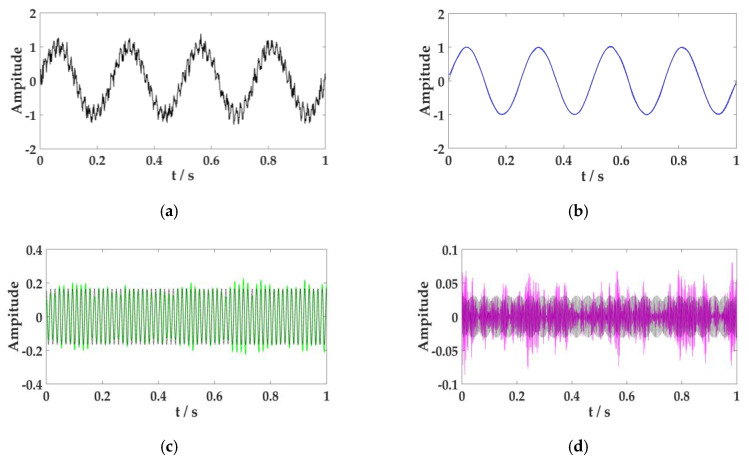
Effect diagram of variational mode decomposition (VMD) processing multi-harmonic signal. (**a**) The composite multi-harmonic signal graph, (**b**) the model component 1 after VMD processing, (**c**) the model component 2 after VMD processing, and (**d**) the model component 3 after VMD processing. The black in the figure is the original signal, and the other colors indicate decomposed signals.

The synthetic signal is composed of three harmonic components. The amplitude of components decreases gradually, whereas the frequency increases gradually. η ~ N(0,σ) is the Gaussian noise. The standard deviation was set to σ = 0.1. Let the number of components of VMD be *K* = 3, the penalty factor be *α* = 2000, the discrimination accuracy be *ε* = 1 × e^−7^, and the fidelity coefficient be *τ* = 0. The result of the signal decomposition is shown in Figure 1.

Figure 1 reveals the low-frequency component 1 and intermediate-frequency component 2 obtained by the VMD processing, which were near to the original signal. The reconstruction effect of the high-frequency signal 3 was unsatisfactory because of the influence of the Gaussian noise.

**Figure 2:**
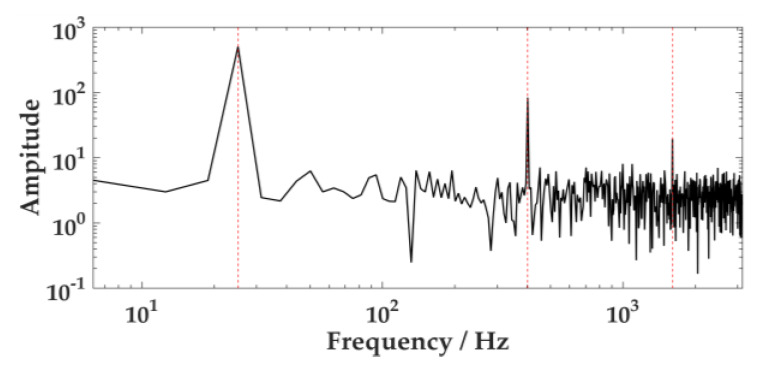
Frequency distribution of noisy multi-harmonic signals.

**Figure 3:**
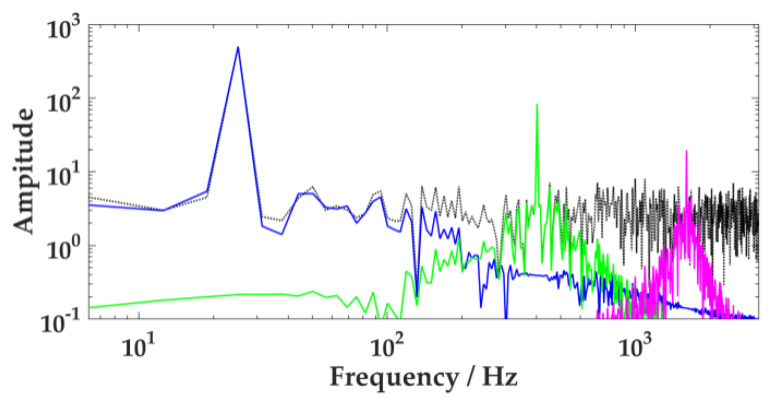
Frequency distribution of variational mode decomposition (VMD) model components.

By comparing Figure 2 and Figure 3, the central frequency of each component can be accurately located after the multi-harmonic signal with the Gaussian noise is processed by VMD.

Research shows *K* and α have more influence on the decomposition of VMD, and ε and τ have no significant effect. Compared with other model decomposition algorithms, VMD must be set to the number of components, *K,* in advance. An improper *K* value will cause over-decomposition or under-decomposition of signals, which will affect the accuracy of the decomposition results. The value of the penalty factor α affects the bandwidth of the model components.

**Figure 4:**
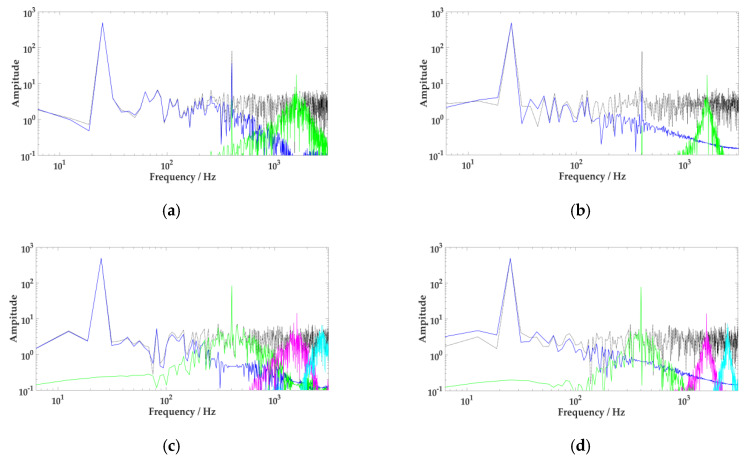
Effect of different *K* and α on variational mode decomposition (VMD). (**a**): *K* = 2, α = 300, (**b**): *K* = 2, α = 3000, (**c**): *K* = 4, α = 300, (**d**): *K* = 4, α = 3000.

In order to show the effect of the number of components, *K,* and penalty factor, α, on the decomposition of VMD, the *K* value should be set to 2 or 4, and the α value should be set as 300 or 3000, respectively, for comparative analysis. The results are demonstrated in Figure 4.

When *K* = 2, the signal is under-decomposed. The number of model components decomposed by VMD is less than the number of the actual value. However, if α = 300, the bandwidth of each component is greater. As described in Figure 4a, when the intermediate frequency component is decomposed into low-frequency ingredients, the issue of model sharing occurs. When α = 3000, the component bandwidth becomes smaller. The intermediate-frequency component is discarded in the decomposition process, as shown in Figure 4b.

When *K* = 4, the signal appears over-decomposed, and the number of model components decomposed by VMD is relatively large. When α = 300, as Figure 4c indicates, the Gaussian noise signal is decomposed into the purple section. If α = 3000, Figure 4d shows how several modes share important frequency components, and false parts appear.

Dissimilar to the synthetic multi-harmonic signal, the collected acoustic signal is more complicated and changeable. How to settle the appropriate *K* and *α* has become the key in analyzing the acoustic signal of the drum shearer using the VMD algorithm. If only one value of *K* or α remains unchanged, VMD is optimized by changing the value of another parameter. Finally, it can only obtain the relative optimal results. Meanwhile, this method also ignores the interaction between the two parameters. As a common swarm intelligence optimization algorithm, PSO has excellent global optimization ability. Therefore, we have chosen to use PSO to optimize the two important parameters of *K* or α of the VMD algorithm.

PSO has an issue in deciding particle fitness function when searching for the parameters of the VMD algorithm. When the particle updates the position, it must recalculate the fitness value once and then update by comparing the fitness value twice. Therefore, the acoustic signals of the drum shearer presents a non-linear and non-stationary signal, which includes a periodic impact signal, harmonic signal, transient signal, and other signals. As a dimensionless index, the peak factor $C_{E}$ of the envelope spectrum considers the strength and periodicity of the shock component in the signal. If there is a signal envelope spectrum amplitude sequence of $X_{j}(j=1,\;2,\;\cdots ,\;M)$, then $C_{E}$ can be calculated from Formula (18):

(18) $$C_{E}=\frac{\max(X_{j})}{\sqrt{\sum_{j}^{M}X_{j}^{2}/M}}$$

where ${max(X}_{i})$ is the maximum value of the envelope spectrum in the frequency range [${n\times f}_{r},\;f_{s}/2$]. Moreover, $f_{r}$ is the rotation frequency of the rotary drive system, and $f_{s}$ is the signal sampling frequency. Experiments have shown that *n* = 3 can avoid the influence of system frequency conversion on the peak factor of the envelope spectrum.

After processing the acoustic signal of the shearer, it obtained some IMFs. If the IMF contains more irrelevant signals and cannot accurately reflect the characteristics of the original signal, it has a smaller $C_{E}$. Supposing that the IMF contains more eigenvalues of the original signal, $C_{E}$ is larger.

In the process of PSO optimizing VMD, when particle *i* is in a specific position, there is a corresponding set of *K* and α. Then, the $C_{E}$ of all IMF components should be calculated after VMD processing. The largest value in the result is defined as the local maximum $C_{E}$. It can be expressed as ${max}_{L}C_{E}^{IMF}$. The IMF corresponding to this value is the local optimal component, which contains sufficient characteristic information of the original signal. To generate the global best component, the best IMF with the most feature information can be obtained by decomposing the original signal. In this paper, ${max}_{L}C_{E}^{IMF}$ will be regarded as the fitness function of optimization with the maximum ${max}_{L}C_{E}^{IMF}$ as the optimization goal.

The steps of VMD based on PSO optimization are as follows:

1 Initialize the parameters of PSO.
2 Determine the fitness function in optimization.
3 Initialize the particle population. A certain number of parameter combinations [*K*, α] are randomly generated as the initial positions of particles. Stochastically initialize the movement speed of each particle.
4 When the particles are in different positions, VMD processes the signals. Count the fitness value ${max}_{L}C_{E}^{IMF}\;$ of the corresponding position of any [*K*, α].
5 Compare the size of fitness values and update $P_{id}$ and $P_{gd}$.
6 Update the velocity and position of particles according to Formulas (11) and (12).
7 If gaining the optimal position or reaching the maximum number of iterations, the optimization ends. Otherwise, repeat steps 4 to 6.

**Figure 5:**
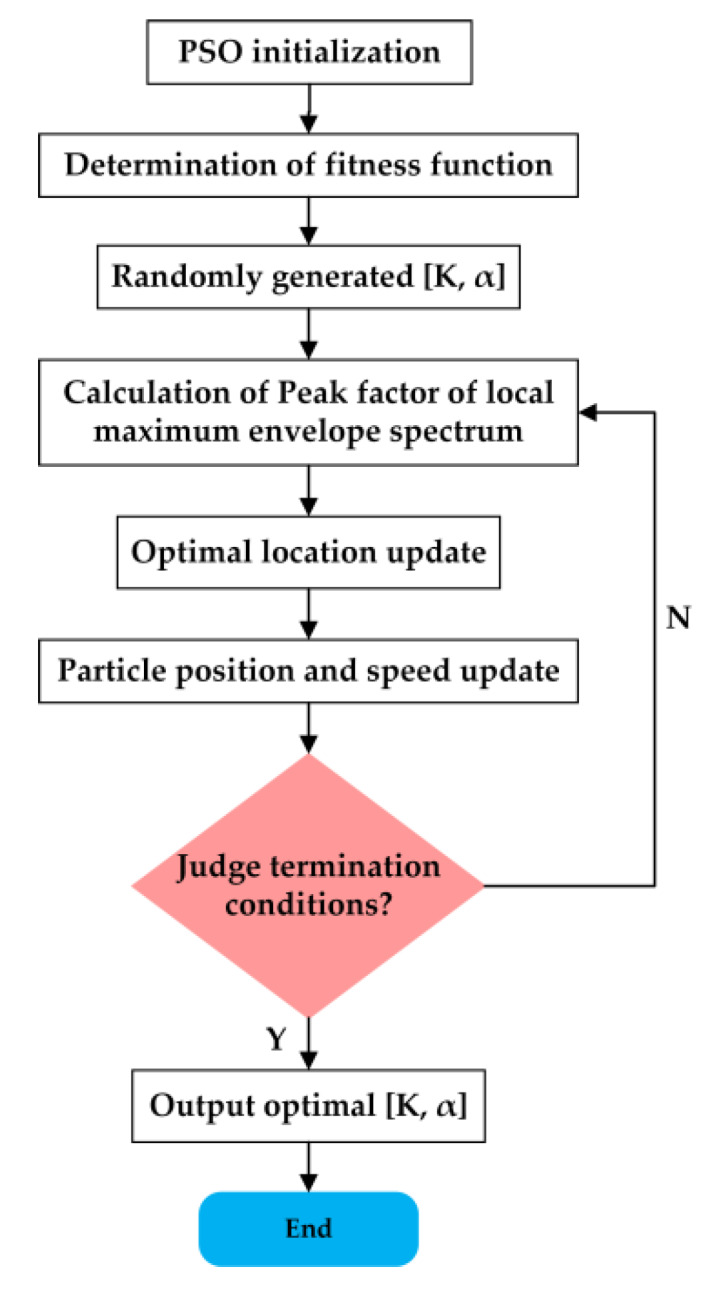
Flow chart of particle swarm optimization (PSO) optimized variational mode decomposition (VMD).

The flow chart of VMD optimization through PSO is described in Figure 5.

## 4. Simulation Signal Analysis

This paper optimizes VMD influence parameters through PSO. To establish a simulation signal for analysis to verify the effectiveness of the proposed approach compare the decomposition effect of this approach with EEMD. The simulation signal is as follows:

(19) $$\left\{ \begin{array}{l} x_{1}(t)=2e^{-100t}\sin(1200\pi t+\frac{\pi }{3})\\ x_{2}(t)=\cos(200\pi t)\\ x_{3}(t)=\sin(64\pi t),t<0.5\\ x(t)=x_{1}(t)+x_{2}(t)+x_{3}(t)+\eta \end{array}\right.$$

where $x_{1}\left(t\right)$ is the periodic impact signal, $x_{2}\left(t\right)$ is the cosine signal, and $x_{3}\left(t\right)$ is the frequency mutation signal. η ~ N(0,σ) is the Gaussian noise, σ = 0.1 In this paper, PSO analyzed the ideal combination of parameters *K* and α of the VMD algorithm. The parameters of the initial PSO algorithm are listed in Table 1. In Table 1, $\omega_{min}$ is the minimum inertia weight coefficient, $\omega_{max}$ is the maximum inertia weight coefficient, *M* is the population size, and $G_{max}$ is the maximum number of iterations.

**Figure 6:**
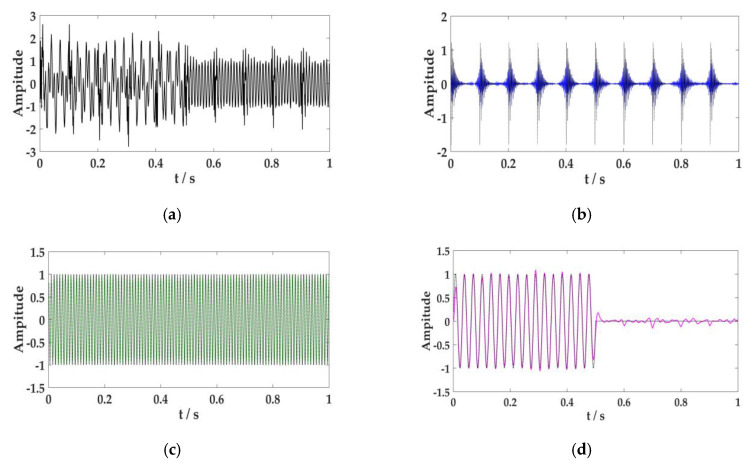
Results of variational mode decomposition (VMD) processing the simulation signal. (**a**) The simulation signal, x(t), (**b**) the periodic impact signal, $x_{1}\left(t\right)$, (**c**) the cosine signal, $x_{2}\left(t\right)$, (**d**) the frequency mutation signal, ${\;x}_{3}\left(t\right)$. The black curve in the figure is the original signal, and other color curves indicate the model components after decomposition.

The optimal parameter combination searched with this approach was [*K*, α] = [3, 1725]. To achieve this combination, the number of model components was set to *K* = 3, and the penalty factor was set to α = 1725. Variational mode decomposition solved the simulation signal. The results are shown in Figure 6. Figure 6a indicates the simulation signal, x(t). Figure 6b–d expresses signals $x_{1}\left(t\right)$, $x_{2}\left(t\right)$, and ${\;x}_{3}\left(t\right)$, respectively. 

**Figure 7:**
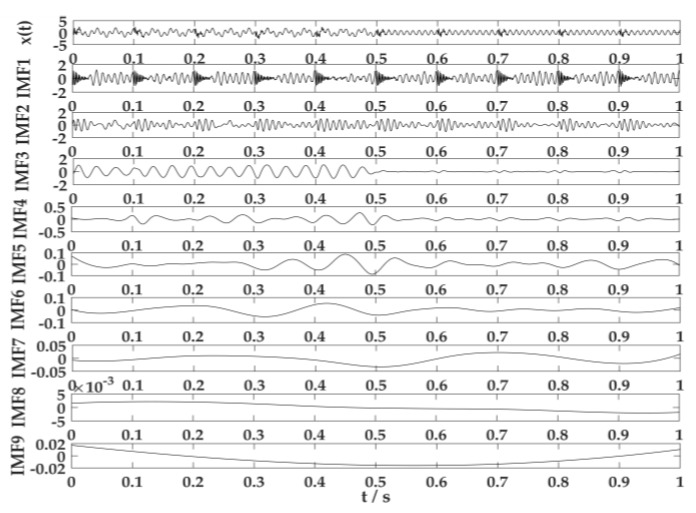
Results of ensemble empirical mode decomposition (EEMD) processing the simulation signal.

Figure 6 illustrates that the non-linear and non-stationary simulation signal can decompose the original signal accurately after VMD processing. The simulation signal by EEMD is processed, and results have been drawn in Figure 7.

Figure 7 explains the simulation signal decomposed into nine ingredients through EEMD processing. Moreover, IMF1 is the separated periodic impact signal $x_{1}\left(t\right)$, and IMF2 corresponds to the cosine signal $x_{2}\left(t\right)$. This aspect is affected by the mode confusion, resulting in a poor decomposition effect. The Mutation signal $x_{3}\left(t\right)$ mostly matched with IMF3, and part of IMF4 was mixed. The rest were false components.

By comparing the effects of the parameter optimized by VMD and EEMD, the VMD algorithm optimized by PSO could accurately decompose the component signals in complicated signals. The decomposition effect of this approach was better than EEMD, which provided assistance for the feature selection and classification of acoustic signals.

Assuming there is a zero-mean signal, $X_{j}(j=1,\;2,\;\cdots ,\;N)$, the envelope signal transforms into a probability distribution sequence in the form of $p_{j}$.

(20) $$p_{j}=\frac{a(j)}{\sum_{j=1}^{N}a(j)}$$

where a(j) is the envelope signal obtained by the Hilbert demodulation, and $p_{j}$ is the normalized form of a(j). The sparsity of the original signal can be determined by calculating the envelope entropy. The calculation formula for signal entropy is as follows:

(21) $$E_{p}=-\sum_{j=1}^{N}p_{j}\mathrm{lg}p_{j}$$

The acoustic signal of the drum shearer is a complex signal and includes a periodic impact signal. If the IMF component contains a significant impact signal, it exhibits strong sparsity, and the value of $E_{p}$ is relatively small [44]. Otherwise, the value is large. Therefore, this paper considers the minimum $E_{p}$ as the signal eigenvalue for classifying the acoustic signal.

## 5. Acoustic Signal Analysis and Classification of the Drum Shearer

**Figure 8:**
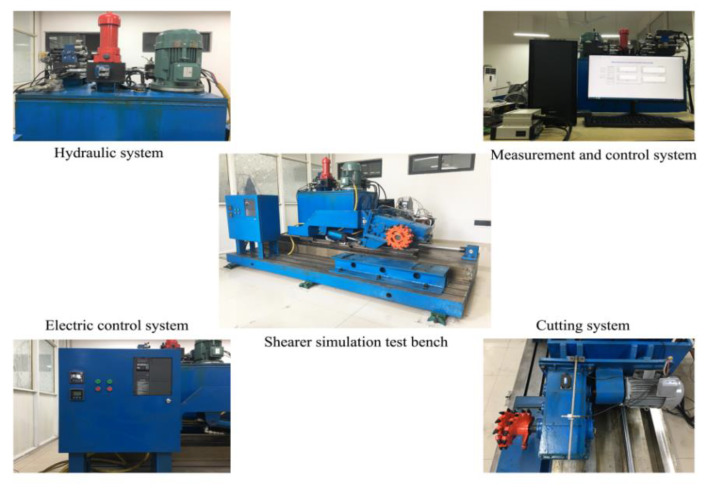
Shearer simulation platform.

In order to verify the effectiveness of the proposed approach for feature extraction and classification, measurements, and analysis of the acoustic signal of the drum shearer are required. Considering the harsh and complicated working environment of coal mines, it is difficult to collect signals on site. Combined with the similarity theory and the MG2X160/710WD shearer, the laboratory has designed a shearer simulation platform, as shown in Figure 8.

### 5.1. Signal Acquisition

**Figure 9:**
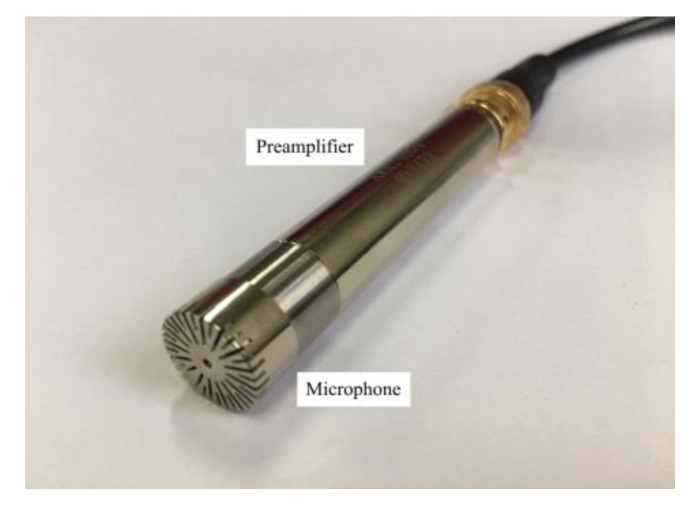
Structure of acoustic sensor.

**Figure 10:**
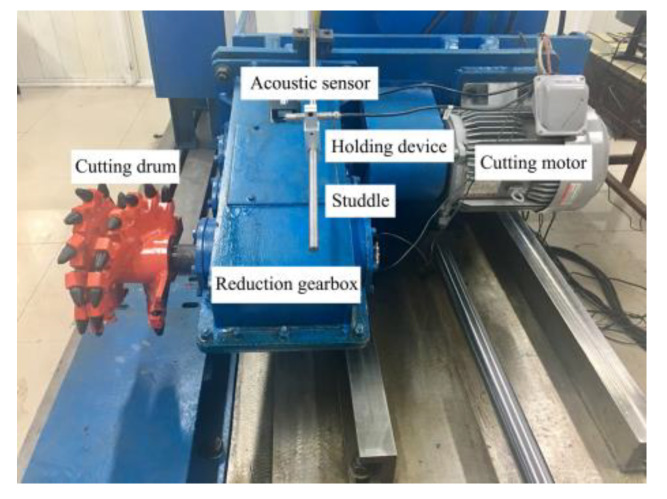
Sensor installation.

The front end of the acoustic sensor is primarily composed of an AWA14420 microphone and an AWA14604 preamplifier, and its structure is shown in Figure 9. The platform put to use the single drum mode. Its rocker arm fixes with a reduction gearbox, where a studdle is arranged above it. The acoustic sensor is fixed on the side toward the cutting roller using the clamping device. The installation of such is described in Figure 10.

The PCIe-6323 data acquisition card of National Instruments is selected for acoustic signal acquisition. Table 2 lists the performance parameters of PCIe-6323. When collecting signals, the acoustic sensor converted the acoustic signal into a voltage signal and transmitted it to the data acquisition card. Then, the signal was changed into a digital signal by analog-to-digital conversion and input to the computer. A signal acquisition and display program was established through LabVIEW 2017.

**Figure 11:**
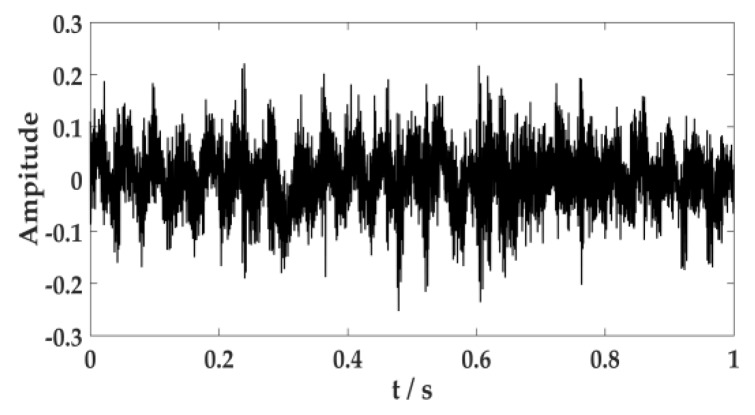
Acoustic signal of the cutting system.

**Figure 12:**
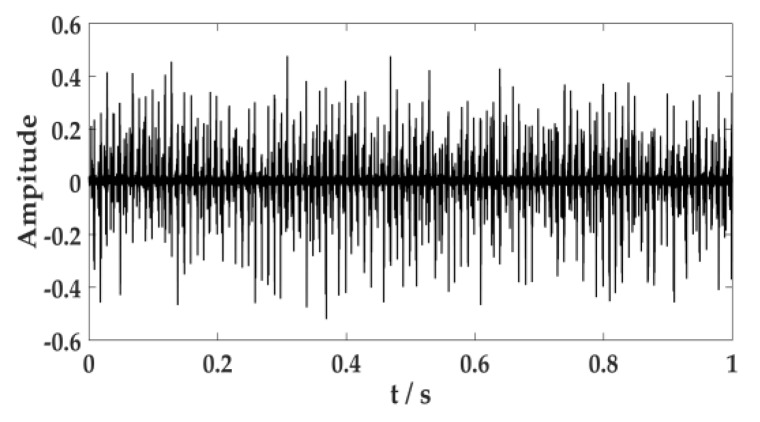
Acoustic signal of the hydraulic system.

Environmental noise, fuselage noise, and random error will cause errors in the collection of acoustic signal. Therefore, it is necessary to ensure the signal is collected multiple times in the same environment. The acoustic signal of the cutting system is often caused by the cutting motor connecting to the reduction gearbox through coupling to drive the cutting drum to rotate. The acoustic signal of the hydraulic system is primarily generated by the pump station motor driving the gear pump. The human ear can distinguish sound frequencies between 20 Hz and 20 KHz. According to the sampling theorem, the sampling frequency should be set to 44.1 KHz, and the sampling time should be set to 1s. The acoustic signal of the cutting system and the hydraulic system are expressed in Figure 11 and Figure 12.

### 5.2. Signal Analysis

**Figure 13:**
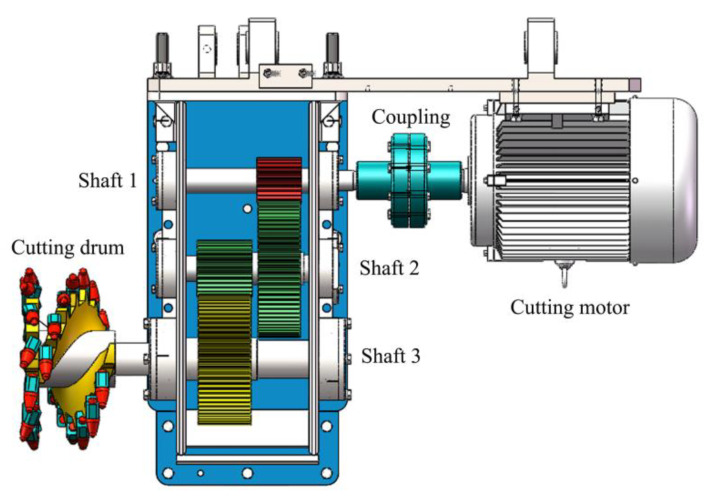
Cutting system structure.

Consider the acoustic signal processing of cutting system as an example. Figure 13 indicates the structure of the cutting system.

As the power source of the cutting system, the cutting motor requires comprehensive attention to the load characteristics, such as power, torque, and speed regulation characteristics. Finally, the designers selected a YX3-132S-4 standard Mitsubishi electric motor. The cutting motor connected with the secondary reduction gearbox through coupling to drive the cutting drum. The working frequency of the motor was then set to 50 Hz. The transmission parameters of the secondary reduction gearbox are listed in Table 3.

**Figure 14:**
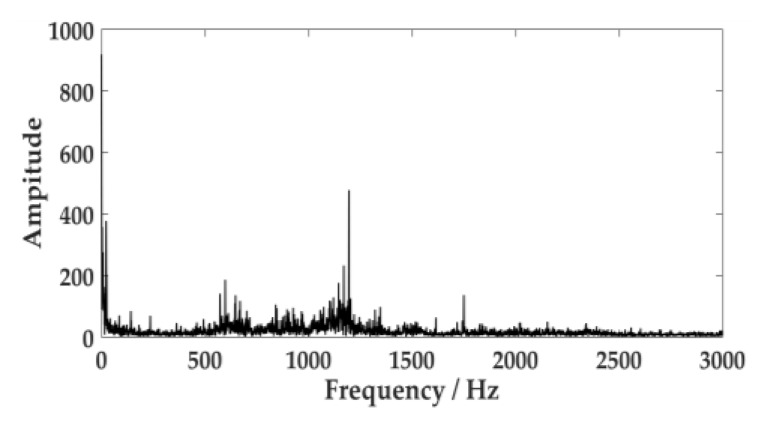
Frequency spectrum of the acoustic signal.

**Figure 15:**
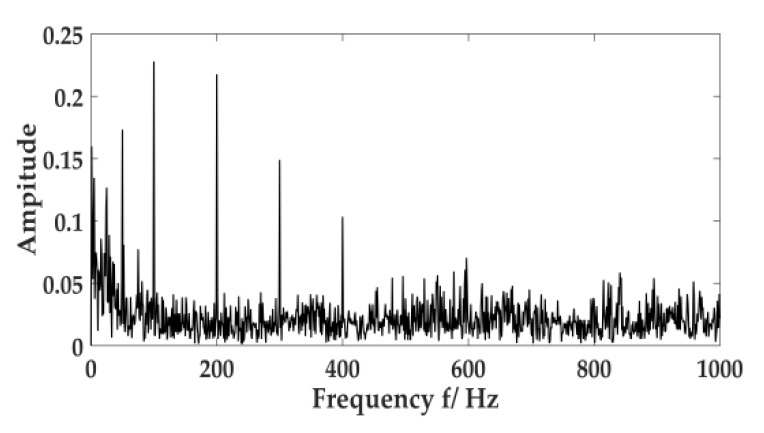
Envelope spectrum of the acoustic signal.

Figure 14 describes the frequency spectrum of the acoustic signal of the cutting system. It displays multiple frequency ingredients in the acoustic signal of the cutting system. The envelope demodulation was performed on the acoustic signal, and the envelope spectrum of the signal is expressed in Figure 15. There are still many spectral peak components in the graph. In this paper, the improved VMD approach would resolve the signal.

**Figure 16:**
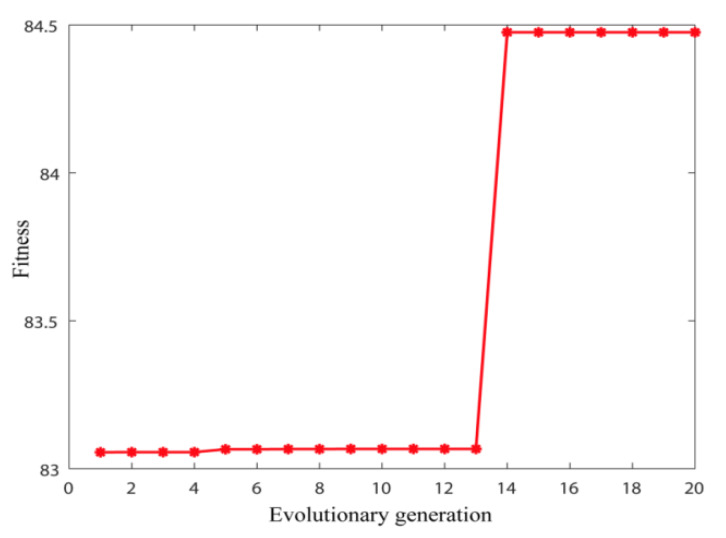
Relationship between fitness value and evolutionary generation.

Figure 16 indicates the change of the ${max}_{L}C_{E}^{IMF}$ with the evolution generation in optimization.

When the particle swarm evolved to the 14th generation, the maximum value of ${max}_{L}C_{E}^{IMF}$ reached 84.492. The output optimal [*K*, α] combination is [14, 100]. The improved VMD algorithm is used to process the acoustic signal of the cutting system. By calculating the envelope entropy of 14 IMF components, the envelope entropy of the second IMF was the lowest, which reached 7.4883. This component contained more abundant signal feature information.

### 5.3. Acoustic Signal Classification of PSO Optimized Clustering Algorithm

Cluster analysis is an unsupervised machine learning method that does not require human annotation and pre-training. During the running time, similar samples will classify into the same category, and dissimilar samples will divide into different categories. To improve the accuracy and speed of clustering analysis, we designed a clustering analysis algorithm through PSO and measured 40 sets of the acoustic signal samples for each of the cutting system and hydraulic system. The PSO-VMD approach interpreted the acoustic signal. Then, we obtained the minimum envelope entropy of the signal component and applied the feature to classify and recognize the acoustic signal. Table 4 lists 80 groups of envelope entropy values.

**Figure 17:**
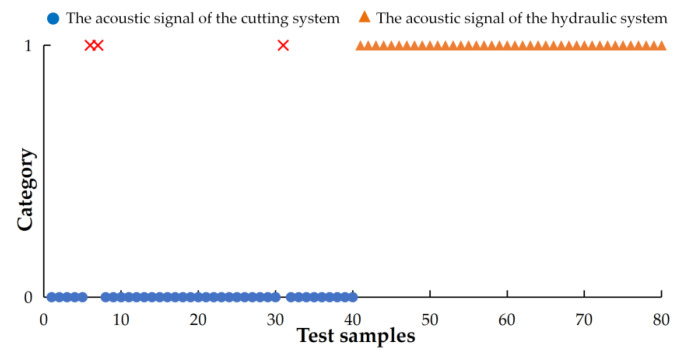
Classification results.

The parameters of the PSO clustering algorithm were as follows: the number of particles was 70; the learning factors were ${\;c}_{1}=1.6$ and ${\;c}_{2}=1.6$; the maximum inertia weight coefficient, $\omega_{max}$, was set to 0.9; the minimum inertia weight coefficient was $\omega_{min}=0.4$; and the maximum number of iterations ${\;G}_{max}$ was 1600. Using this method to classify the acoustic signals, the classification results are expressed in Figure 17.

Through the analysis, it shows there are three incorrect classifications in the 80 test samples, and the accuracy of the classification recognition was 96.25%.

In order to prove the method proposed in this paper is superior to the methods proposed in other existing studies, according to the literature, this paper selected several acoustic signal processing methods based on shearer cutting for comparison and analysis. Wavelet packet transform (WPT)-PNN (probabilistic neural network); the improved EEMD (IEEMD)-PNN; EEMD-VTWNN (variable translation wavelet neural network); EEMD-VTWNN optimized by modified bat algorithm (MBA); and multi-class F-score (MFS)-MEB-SVM were regarded as the reference groups. The results are listed in Table 5.

For the acoustic signal of the shearer, it was hard for WPT to obtain accurate feature information due to the fixed wavelet basis function and decomposition layer. EEMD improved the ability to acquire features because it could adaptively decompose signals. In addition, the strategy based on swarm intelligence optimization algorithm reformed the recognition rate of the classification algorithm. As a whole, the method proposed in this paper had a better recognition accuracy.

The results show the method proposed can effectively identify the acoustic signals produced by the shearer in operation. In fact, the underground environment is poor during coal mining. Compared with the signals such as vibration and vision, the acoustic signal can be easily obtained and analyzed. The proposed acoustic signal processing method can be used to identify the interface between coal and rock when the drum shearer is cutting, which improves the mining efficiency. In addition, the operating status of the shearer can be dynamically monitored, which helps to increase the service life of the machine.

## 6. Conclusions and Prospects

In this paper, we have proposed an acoustic signal processing approach with PSO-VMD. PSO searched the optimal parameters of VMD, and then the acoustic signal was decomposed. After that, the minimum envelope entropy of the component signal was taken as the feature. Using the PSO-optimized clustering algorithm to classify and recognize the acoustic signal of the drum shearer, the simulation analysis and experimental results indicated that the approach proposed in this paper could effectively process the acoustic signal of the shearer and precisely classify it. Moreover, the accuracy of the acoustic signal classification reached 96.25%.

However, there are still some difficulties in acoustic signal processing and classification. The initial parameter setting of the PSO algorithm lacks rigorous theoretical analysis and derivation and is essentially determined through a large number of simulations. It takes a long time for PSO to ascertain the parameter combination of VMD, which remains in the laboratory research stage. Therefore, in order to quickly and accurately realize the processing and recognition of the acoustic signal during shearer cutting, the authors will perform theoretical analyses and experimental verification on several aspects. This includes the selection of VMD parameters, the efficiency of swarm intelligence optimization algorithms, and the adaptability of online industrial identification systems.

## Figures and Tables

**Table 1:** Initial parameters of particle swarm optimization (PSO).

$c_{1}$	$c_{2}\;$	$\omega_{\min}$	$\omega_{\max}$	*M*	$G_{\max}$
1.5	1.5	0.4	0.9	20	20

**Table 2:** The performance parameters of PCIe-6323.

Performance Parameter	Index Value
Number of analog input channels	16 differential or 32 single ended
Analog–digital converter resolution	16 bits
Single channel maximum sampling rate	250 kS/s
Number of analog output channels	4 channels
Digital–analog converter resolution	16 bits
Signal channel maximum updated rate	900 kS/s

**Table 3:** Transmission parameters of the secondary reduction gearbox.

Transmission Parameters	High-Speed Gear Set	Low-Speed Gear Set
Gear modulus	3	3
Number of pinion teeth	24	30
Number of large gear teeth	76	73
Transmission ratio	3.166	2.433
Pressure angle (°)	20	20

**Table 4:** Envelope entropy of feature.

Experience Group	Optimal Envelope Entropy
1	[7.4883, 7.4443, 7.3487, 7.4516, 7.5074, 7.7370, 7.9055, 7.5466, 7.3212, 7.3465, 7.3406, 7.2994, 7.4831, 7.4870, 7.2313, 7.4551, 7.6440, 7.3354, 7.4853, 7.4946, 7.5568, 7.4802, 7.4539, 7.4824, 7.3477, 7.4989, 7.3541, 7.5143, 7.4427, 7.5808, 7.8402, 7.5027, 7.2932, 7.3216, 7.6472, 7.4759, 7.4542, 7.4661, 7.4849, 7.2856]
2	[8.0925, 7.9822, 8.0960, 8.1214, 8.0436, 7.9766, 8.0436, 7.9983, 7.9526, 7.9896, 8.0836, 8.1699, 7.9583, 7.9885, 8.0601, 8.1000, 7.9492, 7.9937, 8.1170, 8.0792, 7.9660, 8.1463, 8.1404, 7.9877, 8.0229, 8.0735, 8.0397, 8.0138, 7.9815, 7.9750, 8.0153, 8.0927, 8.0774, 8.0119, 7.9693, 8.0375, 8.0345, 7.9721, 7.9597, 8.0176]

**Table 5:** Comparison between different recognition methods.

Compared Methods	Recognition Accuracy
WPT-PNN	78.33%
EEMD-VTWNN	84.75%
IEEMD-PNN	92.67%
MFS-MEB-SVM	94.42%
EEMD-VTWNN-MBA	95.25%
The proposed method	96.25%

## References

[1] Yang H., Li W., Luo C., Zhang J., Si Z. (2016). Research on Error Compensation Property of Strapdown Inertial Navigation System Using Dynamic Model of Shearer. IEEE Access.

[2] Bessinger S.L., Neison M.G. (1993). Remnant roof coal thickness measurement with passive gamma ray instruments in coal mine. IEEE Trans. Ind. Appl..

[3] Fan S.Z., Geng M.X., Xu J.P. (1996). The application of pattern recognition in the automatic vertical steering system of shearer’s drum. Intel. J. Coal. Sci. Technol..

[4] Sun J., Su B. (2013). Coal–rock interface detection on the basis of image texture features. Int. J. Mining Sci. Technol..

[5] Zhang Q., Wang H.J., Wang Z., Wen X.Z. (2016). Analysis of Coal—Rock’s Cutting Characteristics and Flash Temperature for Peak Based on Infrared Thermal Image Testing. Chin. J. Sens. Actuators.

[6] Xu J., Wang Z., Tan C., Si L., Liu X. (2015). A cutting pattern recognition method for shearers based on improved ensemble empirical mode decomposition and a probabilistic neural network. Sensors.

[7] Xu J., Wang Z., Tan C., Si L., Liu X. (2018). Cutting pattern identification for coal mining shearer through a swarm intelligence–based variable translation wavelet neural network. Sensors.

[8] Si L., Wang Z., Liu X., Tan C. (2019). A sensing identification method for shearer cutting state based on modified multi-scale fuzzy entropy and support vector machine. Eng. Appl. Artif. Intell..

[9] Song Q.J., Jiang H., Song Q.H., Zhao X.G., Wu X.X. (2017). Combination of minimum enclosing balls classifier with SVM in coal-rock recognition. PLoS ONE.

[10] Song Q., Jiang H., Zhao X., Li D. (2017). An automatic decision approach to coal–rock recognition in top coal caving based on MF-Score. Pattern Anal. Appl..

[11] Li J., Yue J., Yang Y., Zhan X., Zhao L. (2017). Multi-Resolution Feature Fusion model for coal rock burst hazard recognition based on Acoustic Emission data. Measurement.

[12] Wang H., Zhang Q. (2019). Dynamic identification of coal-rock interface based on adaptive weight optimization and multi-sensor information fusion. Inform. Fusion.

[13] Lazarev I.E., Sayfulina K.E., Chernysheva E.G., Bryzgalov D.V., Chernyshev B.V. (2018). Feature binding in auditory modality requires attention as indexed by mismatch negativity and N2b in an active discrimination task. NeuroReport.

[14] Jiménez-Fernández A., Cerezuela-Escudero E., Miró-Amarante L., Domínguez-Morales M.J., de Asís Gómez-Rodríguez F., Linares-Barranco A., Jiménez-Moreno G. (2016). A binaural neuromorphic auditory sensor for FPGA: A spike signal processing approach. IEEE. Trans. Neur. Net. Lear. Syst..

[15] Ingerman E.A., London R.A., Heintzmann R., Gustafsson M.G.L. (2019). Signal, noise and resolution in linear and nonlinear structured-illumination microscopy. J. Microsc..

[16] Goyal B., Dogra A., Agrawal S., Sohi B.S., Sharma A. (2020). Image denoising review: From classical to state-of-the-art approaches. Inform. Fus..

[17] Wang P., Fu H., Zhang K. (2018). A pixel-level entropy-weighted image fusion algorithm based on bidimensional ensemble empirical mode decomposition. Int. J. Distrib. Sens. Netw..

[18] Zhang N., Lin A., Shang P. (2017). Multidimensional k-nearest neighbor model based on EEMD for financial time series forecasting. Phys. A Stat. Mech. Appl..

[19] Wang Z., Wang J., Cai W., Zhou J., Du W., Wang J., He G., He H. (2019). Application of an improved ensemble local mean decomposition method for gearbox composite fault diagnosis. Complexity.

[20] Yan R., Gao R.X., Chen X. (2014). Wavelets for fault diagnosis of rotary machines: A review with applications. Signal Process..

[21] Huang N.E., Wu Z. (2008). A review on Hilbert-Huang transform: Method and its applications to geophysical studies. Rev. Geophys..

[22] Klionskiy D., Kupriyanov M., Kaplun D. (2017). Signal denoising based on empirical mode decomposition. J. Vibroeng..

[23] Wang S., Zhang N., Wu L., Wang Y. (2016). Wind speed forecasting based on the hybrid ensemble empirical mode decomposition and GA-BP neural network method. Renew. Energ..

[24] Zhang X., Liang Y., Zhou J. (2015). A novel bearing fault diagnosis model integrated permutation entropy, ensemble empirical mode decomposition and optimized SVM. Measurement.

[25] Yang Y., Cheng J., Zhang K. (2012). An ensemble local means decomposition method and its application to local rub-impact fault diagnosis of the rotor systems. Measurement.

[26] Dragomiretskiy K., Zosso D. (2013). Variational mode decomposition. IEEE. Trans. Signal. Process..

[27] Wang Y., Markert R., Xiang J., Zheng W. (2015). Research on variational mode decomposition and its application in detecting rub-impact fault of the rotor system. Mech. Syst. Signal. Process..

[28] Li Z., Chen J., Zi Y., Pan J. (2017). Independence-oriented VMD to identify fault feature for wheel set bearing fault diagnosis of high speed locomotive. Mech. Syst. Signal. Process..

[29] Yan X., Jia M., Xiang L. (2016). Compound fault diagnosis of rotating machinery based on OVMD and a 1.5-dimension envelope spectrum. Meas. Sci. Technol..

[30] Yi C., Lv Y., Dang Z. (2016). A Fault Diagnosis Scheme for Rolling Bearing Based on Particle Swarm Optimization in Variational Mode Decomposition. Shock Vib..

[31] Zhang X., Miao Q., Zhang H., Wang L. (2018). A parameter-adaptive VMD method based on grasshopper optimization algorithm to analyze vibration signals from rotating machinery. Mech. Syst. Signal Process..

[32] Yan X., Jia M. (2019). Application of CSA-VMD and optimal scale morphological slice bispectrum in enhancing outer race fault detection of rolling element bearings. Mech. Syst. Signal Process..

[33] Zheng X., Wang S., Qian Y. (2019). Fault feature extraction of wind turbine gearbox under variable speed based on improved adaptive variational mode decomposition. Proc. Inst. Mech. Eng. Part J. Pow. Eng..

[34] Mok P.Y., Huang H.Q., Kwok Y.L., Au J.S. (2012). A robust adaptive clustering analysis method for automatic identification of clusters. Pattern Recognit..

[35] Ahmad A., Hashmi S. (2016). K-Harmonic means type clustering algorithm for mixed datasets. Appl. Soft Comput..

[36] Baraldi P., Di Maio F., Rigamonti M., Zio E., Seraoui R. (2015). Clustering for unsupervised fault diagnosis in nuclear turbine shut-down transients. Mech. Syst. Signal Process..

[37] Tang J., Zhang G., Wang Y., Wang H., Liu F. (2015). A hybrid approach to integrate fuzzy C-means based imputation method with genetic algorithm for missing traffic volume data estimation. Transp. Res. Part Emerg. Technol..

[38] Biniaz A., Abbasi A. (2014). Unsupervised ACO: Applying FCM as a supervisor for ACO in medical image segmentation. J. Intell. Fuzzy Syst..

[39] Guofeng J., Wei Z., Zhengwei Y., Zhiyong H., Yuanjia S., Dongdong W., Gan T. (2012). Image segmentation of thermal waving inspection based on particle swarm optimization fuzzy clustering algorithm. Meas. Sci. Rev..

[40] Duan H., Luo Q. (2015). New progresses in swarm intelligence–based computation. Int. J. Bio Inspir. Comp..

[41] Yahya A.A., Osman A., El-Bashir M.S. (2017). Rocchio algorithm-based particle initialization mechanism for effective PSO classification of high dimensional data. Swarm Evol. Comput..

[42] Malik S., Kim D.H. (2018). Prediction-learning algorithm for efficient energy consumption in smart buildings based on particle regeneration and velocity boost in particle swarm optimization neural networks. Energies.

[43] Maleki A., Ameri M., Keynia F. (2015). Scrutiny of multifarious particle swarm optimization for finding the optimal size of a PV/wind/battery hybrid system. Renew. Energ..

[44] Tang G.J., Wang X.L. (2015). Parameter Optimized Variational Mode Decomposition Method with App-lication to Incipient Fault Diagnosis of Rolling Bearing. Chin. J. Xi’An Jiaotong Univ..

